# HPLC Determination of Imidazoles with Variant Anti-Infective Activity in Their Dosage Forms and Human Plasma

**DOI:** 10.3390/molecules26010129

**Published:** 2020-12-30

**Authors:** Oday T. Ali, Wafaa S. Hassan, Ahdab N. Khayyat, Ahmad J. Almalki, Mahmoud M. Sebaiy

**Affiliations:** 1 Department of Chemistry, Faculty of Science, Zagazig University, 44519 Zagazig, Egypt; odyali95@gmail.com; 2 Ministry of Education, Baghdad 55509, Iraq; 3 Department of Analytical Chemistry, Faculty of Pharmacy, Zagazig University, 44519 Zagazig, Egypt; bellal_elmadina@yahoo.com; 4 Department of Pharmaceutical Chemistry, Faculty of Pharmacy, King Abdulaziz University, Jeddah 21589, Saudi Arabia; ankhayyat@kau.edu.sa (A.N.K.); ajalmalki@kau.edu.sa (A.J.A.); 5 Department of Medicinal Chemistry, Faculty of Pharmacy, Zagazig University, 44519 Zagazig, Egypt

**Keywords:** HPLC, anti-infective, imidazole, dosage forms, human plasma

## Abstract

A suitable HPLC method has been selected and validated for rapid simultaneous separation and determination of four imidazole anti-infective drugs, secnidazole, omeprazole, albendazole, and fenbendazole, in their final dosage forms, in addition to human plasma within 5 min. The method suitability was derived from the superiority of using the environmentally benign solvent, methanol over acetonitrile as a mobile phase component in respect of safety issues and migration times. Separation of the four anti-infective drugs was performed on a Thermo Scientific^®^ BDS Hypersil C_8_ column (5 µm, 2.50 × 4.60 mm) using a mobile phase consist of MeOH: 0.025 M KH_2_PO_4_ (70:30, *v/v*) adjusted to pH 3.20 with ortho-phosphoric acid at room temperature. The flow rate was 1.00 mL/min and maximum absorption was measured with UV detector set at 300 nm. Limits of detection were reported to be 0.41, 0.13, 0.18, and 0.15 µg/mL for secnidazole, omeprazole, albendazole, and fenbendazole, respectively, showing a high degree of the method sensitivity. The method of analysis was validated according to Food and Drug Administration (FDA)guidelines for the determination of the drugs, either in their dosage forms with highly precise recoveries, or clinically in human plasma, especially regarding pharmacokinetic and bioequivalence studies.

## 1. Introduction

**Figure 1:**
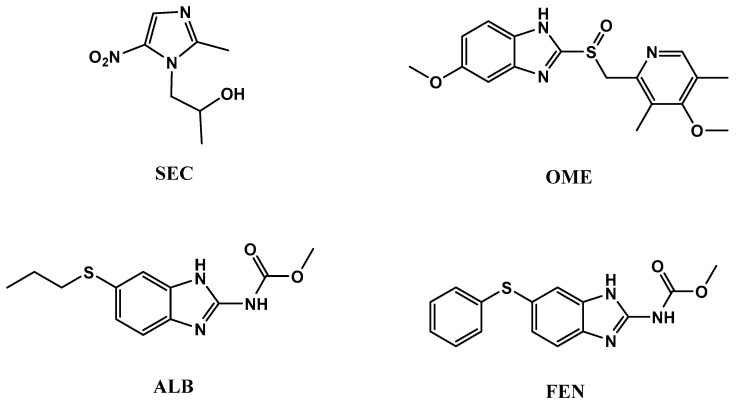
Chemical structures ofsecnidazole (SEC), omeprazole (OME), albendazole (ALB), and fenbendazole (FEN).

One of the main reasons for high mortality rate around the world are infectious diseases. Given the broad scope of infectious diseases that affect men, we have selected some topics that are especially relevant to this patient population, such as protozoa and bacteria [1,2,3,4]. Four imidazole anti-infective drugs with different biological activities were subjected to chemical analysis. Secnidazole (SEC), which is chemically, 1-(2-methyl-5-nitroimidazol-1-yl)propan-2-ol, is categorized as one of the medicinal drugs having 5-nitroimidazole group. It has an anti-protozoal action for dealing of infectious diseases initiated by amoeba, trichomonas, lamblia, leishmania, etc. [5,6]. Omeprazole (OME), 5-methoxy-2-[[(4-methoxy-3,5-dimethyl-2-pyridinyl)methyl]sulphinyl]-1*H*-benzimidazole, is the earliest proton pump inhibitor drug used for the prophylaxis and treatment of both gastro-duodenal ulcers and symptomatic gastroesophageal reflux disease GERD caused by *Helicobacter pylori* bacteria [5,7]. Albendazole (ALB), methyl-[(5-propyl- thio)-1*H*-benzimidazol-2-yl] carbamate and fenbendazole (FEN), methyl-[5-(phenylsulfanyl)-1*H*-benzoim idazol-2-yl] carbamate are used as anthelmintics for the treatment of both the immature and mature stages of nematodes and cistodes parasite of the gastrointestinal and respiratory tracts of humans, sheep, and cattle [5,8,9]. All four chemical structures are represented in Figure 1.

Our literature survey indicated that various analytical techniques have been employed for the estimation of SEC, OME, ALB, and FEN such as UV-vis spectrophotometry [6,7,8,9], HPLC [10,11,12,13], HPTLC [14,15], LC/MS [16,17,18,19], capillary electrophoresis [20,21,22,23] and voltammetric methods [24,25,26,27].

To the best of our knowledge and comprehensive survey, only two reported methods described the chromatographic determination of ALB and FEN [28], and SEC, and OME, together with amoxicillin [29]. However, the four drugs together were not determined before by chromatographic techniques in pharmaceutical or biological samples despite their synergistic action. This mixture of the four drugs is usually prescribed for many patients in Egypt due to the evolution of gastrointestinal GIT infection specifically GERD, amoebiasis, and bilharziasis. In addition, reported HPLC methods [10,11,12,13] had some limitations, especially in run time that was too long, up to 18 min for some drugs. As such, the present work introduces a simple, rapid, reproducible, and sensitive chromatographic method for the determination of the four drugs in both matrices.

## 2. Results and Discussion

### 2.1. Optimization of Chromatographic Conditions

**Figure 2:**
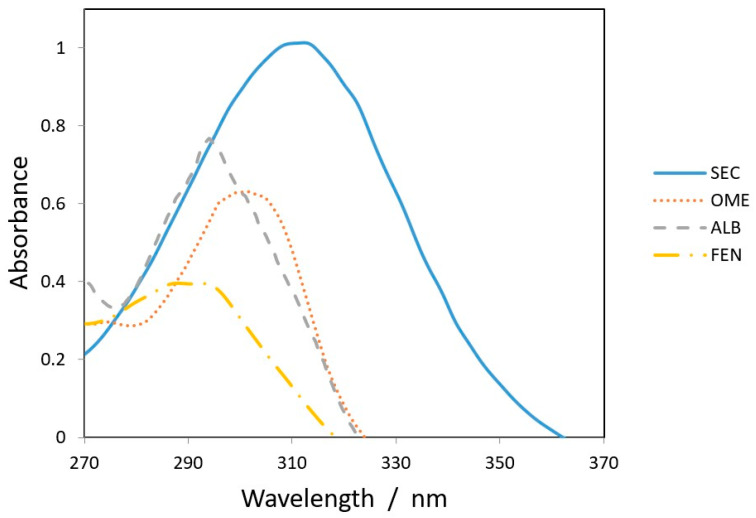
Overlain spectra of 25 µg/mL (SEC___), (OME.....), (ALB_ _ _ _) and (FEN _._._.) at 312, 301, 294, and 288 nm, respectively.

**Figure 3:**
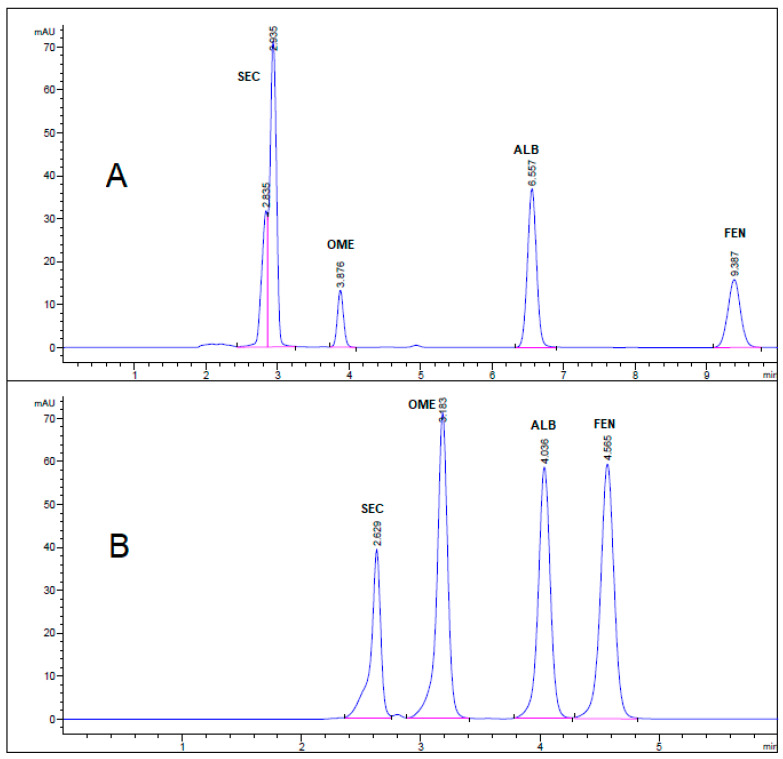
HPLC chromatogram of authentic mixture containing 25 µg/mL SEC, OME, ALB, and FEN using mobile phase (**A**) acetonitrile: 0.025 M KH_2_PO_4_ (60:40, *v/v*) buffer, pH 3.20 and (**B**) methanol: 0.025 M KH_2_PO_4_ (70:30, *v/v*) buffer, pH 3.20. Other chromatographic conditions are stated in Table 1.

**Figure 4:**
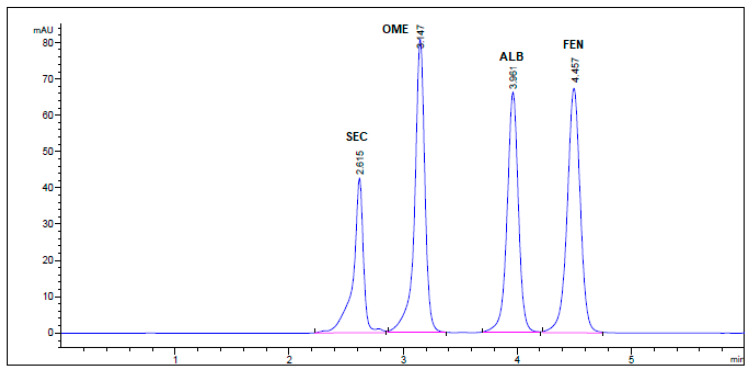
HPLC Chromatogram of Secnidazole^®^, Omez^®^, Alzental^®^, and Curafluke^®^ dosage forms. All optimum chromatographic conditions are stated in Table 1.

**Figure 5:**
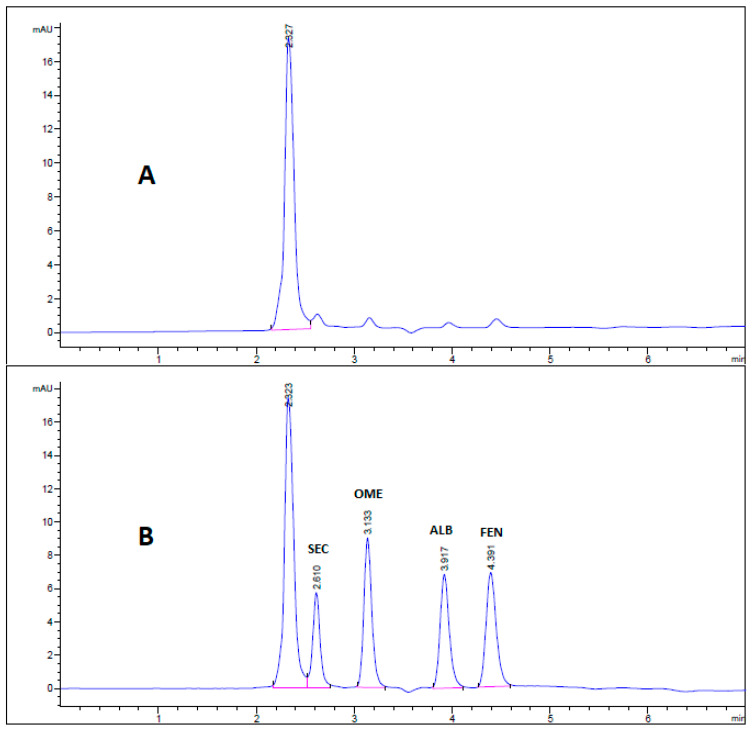
HPLC chromatogram of (**A**) blank human plasma sample, and (**B**) mixture of 1 µg/mL SEC, OME, ALB, and FEN in human plasma sample. All optimum chromatographic conditions are stated in Table 1.

All chromatographic conditions are illustrated in Table 1. The spectroscopic analysis of the four anti-infective drugs, SEC, OME, ALB, and FEN, in the range between 200 and 400 nm, demonstrated a maximum UV absorbance (λ_max_) at 312, 301, 294, and 288 nm respectively as shown in Figure 2. Accordingly, DAD detector was set to measure the absorbance at 300 nm. Furthermore, regarding mobile phase optimization, trials using acetonitrile ACN were not taken in consideration due to safety concerns associated with the use of acetonitrile, lower resolution power, and longer retention times (specially for SEC) in comparison to methanol based mobile phase as shown in Figure 3A. Therefore, methanol: 0.025 M KH_2_PO_4_ (70:30, *v/v*) was selected as the mobile phase for the HPLC method. Following these circumstances, both the tablet dosage form and the pure form of the four anti-infective drugs accomplished, complete baseline separation at 2.63, 3.18, 4.03, and 4.56 min for SEC, OME, ALB, and FEN respectively, as illustrated in Figure 3B and Figure 4. Next, the technique was then used for determination of the anti-infective drugs in human plasma using protein precipitation technique. This technique was preferred than liquid–liquid extraction, where methanol was used for drugs extraction instead of other toxic organic solvents like chloroform, dichloromethane, and petroleum ether beside avoidance of sample loss accompanied with liquid–liquid extraction technique [30]. For the previous reasons, plasma precipitation technique was favored over liquid–liquid extraction (LLE) method. No matrix interference effect was noticed in correspondence to the elution of the analyte of interests, SEC, OME, ALB, and FEN, as blank human plasma elute at 2.37 min (Figure 5A,B). Nevertheless, the mobile phase exhibited a resolution >2, theoretical plates >2000, capacity factor (1 < k < 10), symmetrical peaks (T ≤ 2) and these results are acceptable according to Center for Drug Evaluation and Research CDER recommendations [31]. Table 2 summarize system suitability parameters of the suggested HPLC-DAD method for simultaneous determination of the four anti-infective drugs in both pure and plasma samples.

### 2.2. Method Validation

The method validation was performed according to Food and Drug Administration (FDA) and international conference of harmonization guidelines (ICH) [32,33,34].

#### 2.2.1. Linearity

**Figure 6:**
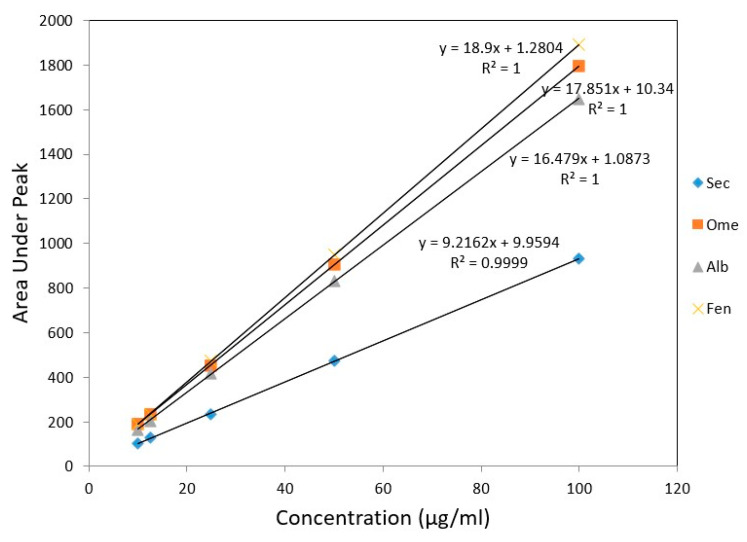
Calibration curves for authentic mixture of SEC, OME, ALB, and FEN using the proposed HPLC method.

For linearity studies, five various concentration of drug mixture were used. The peak area versus concentration showed a linear calibration curve for all drugs in the concentration range of 10–100 µg/mL (Table 3). Linear regression equations of SEC, OME, ALB, and FEN were found to be y = 9.21x + 9.95, y = 17.85x + 10.34, y = 16.47x + 1.08 and y = 18.90x + 1.28, respectively. The calculated regression coefficient values (r) were indicating a high degree of linearity, one for all drugs except for SEC, which was 0.999 (Figure 6).

#### 2.2.2. Accuracy

For the accuracy of the method, standard addition technique with different concentrations within the range were used to evaluate the recoveries of commercial formulations (each concentration triplicate). The percentage recovery was calculated from the amount of the drug estimated by spiking each drug at different levels followed by the suggested method. The findings indicate outstanding recoveries for all drugs (Table 4).

#### 2.2.3. Precision

The precision was calculated to validate the method. This was expressed as standard deviation (SD) and coefficient of variation (CV%) and was computed by analyzing three varying concentrations 25, 50, and 100 µg/mL. Assessment of intra-day precision was determined from the results of triplicate analysis using the same solution containing pure drugs. The SD values (varied from 0.02 to 0.49) and CV% values (varied from 0.04 to 1.96). For inter-day reproducibility, the day-to-day SD and CV% values were also in the acceptable range of 0.06–1.40 and 0.24–2.75, respectively. Table 5 lists the data obtained from intra-day and inter-day precision, which revealed the high precision of the method in simultaneous determination of the four drugs in their pharmaceutical formulations.

#### 2.2.4. Selectivity and Specificity

No interfering peaks were observed by injecting SEC, OME, ALB, and FEN into the column individually. Four well-resolved peaks were attained at retention times of 2.63, 3.18, 4.03, and 4.56 min, respectively, but not attained in the blank solution. The specificity findings confirm the lack of interference from co-eluting excipients in the tablet formulations with the sharp and well-resolved peaks of the four drugs (Figure 4).

#### 2.2.5. Limits of Detection and Limits of Quantification

In order to determine the limits of detection and quantitation, an approach followed based on signal-to-noise ratio (3:1 for limits of detection (LOD) and 10:1 for limits of quantification (LOQ)). Table 3 showed limits of detection for SEC, OME, ALB, and FEN to be 0.41, 0.13, 0.18, and 0.15 µg/mL, respectively. Furthermore, the calculated limits of quantification were be 1.37, 0.44, 0.61, and 0.51 µg/mL, respectively. These satisfactory results, signifies that this method is highly sensitive and applicable in studies which require a detection of small concentrations in plasma as in pharmaceutical analysis, pharmacokinetic, and bioequivalence studies.

#### 2.2.6. Robustness

A minor intentional variation in the flow rate, mobile phase composition ratio, and pH of the mobile phase by ± 0.05 while maintaining the other chromatographic conditions persistent showed a negligible influence on the results of all drugs based on percent recovery and standard deviation (Table 6).

### 2.3. Applications

#### 2.3.1. Analysisof Pharmaceutical Formulations

Secnidazole^®^, Omez^®^, Alzental^®^ and Curafluke^®^ pharmaceutical formulations having SEC, OME, ALB, and FEN, respectively, had been effectively studied by the designed method without any interference with excipients and impurities showing a high level of specificity for the method. Calculated Student’s *t*-test and F-test were used to compare outcomes acquired by the proposed method to those acquired by applying reference methods [10,11,13]. Results showed no statistical significant difference between suggested method and reference ones relative to precision and accuracy (Table 7). Calculated t and F values were less than presented ones for SEC, OME, ALB, and FEN.

#### 2.3.2. Analysis of Human Plasma

Protein precipitation procedure were used to check the clinical applicability of the method for SEC, OME, ALB, and FEN determination in human plasma samples. The system suitability parameters as well as the retention times in plasma samples were alike to those in pure and pharmaceutical formulas (Table 2). The linear calibration curves obtained over the clinical range of 1.00–15.00 μg/mL for the four drugs in the spiked plasma (Table 8). The specificity of the method in the clinical studies confirmed by the plasma chromatogram (Figure 5A,B) as the plasma peak (eluting at 2.37 min) is not interfering with all peaks of SEC, OME, ALB, and FEN. Table 9 summarized a 24 h- room temperature stability and plasma freeze-thaw cycles at −20 °C over three days by using validation QC samples at concentrations of 1.00, 5.00 and 15.00 µg/mL of SEC, OME, ALB, and FEN in plasma. The recoveries for SEC, OME, ALB, and FEN were in the range of 98.18–103.72%, 95.71–98.39%, 93.38–95.67%, and 87.24–89.70%, where coefficients of variation were in the range of 0.58–12.17%, 0.29–4.10, 0.33–3.17%, and 0.01–4.52%, respectively. In terms of sensitivity, LOD, LOQ, and even migration time, the recommended method has been exceeding the formerly stated methods in the studying of SEC, OME, ALB, and FEN in pure samples or in pharmaceutical formulations [10,11,13].

## 3. Experimental

### 3.1. Apparatus

Agilent 1200^®^ HPLC instrument (Germany) with a Thermo Scientific^®^ BDS Hypersil C_8_ column (5 µm, 2.50 × 4.60 mm), DAD absorbance detector, HPLC QUAT pumps and connected to PC computer loaded with Agilent 1200 software.

Jenway^®^ 6800 Spectro UV-VIS Double Beam Spectrophotometer (Chelmsford, UK) with matched 1 cm quartz cells and connected to windows compatible computer uploaded with Flight Deck 1.0 Software.

HANNA^®^ HI 8314 membrane pH-meter (Cluj County, Romania) was for pH adjustment.

### 3.2. Materials and Reagents

All solvents and reagents were of an HPLC analytical grade (methanol, acetonitrile, potassium dihydrogen phosphate, and ortho-phosphoric acid were supported from Fisher Scientific, Loughborough, England).

SEC, OME, ALB, and FEN were kindly provided by different Egyptian companies like Egyptian Company for Pharmaceutical & Chemical Industries (EIPICO) and Pharaonia Pharmaceutical Company (PPC). Stock Standard solutions (200 µg/mL) of each pure drug were made by weighing 20 mg of each pure drug and dissolving in 100 mL of the mobile phase.

Mobile phaseconsisted of MeOH: 0.025 M potassium dihydrogen phosphate (70:30, *v/v*) adjusted to pH 3.20 using ortho-phosphoric acid. The mobile phase was filtered through a 0.45 μm membrane (Millipore, Burlington, USA) then degassed at the time of analysis. Other mobile phase compositions are stated in Results and Discussion section.

Secnidazole^®^ tablets (EIPICO, 10th of Ramadan City, Egypt), Omez^®^ tablets (PPC, Borg Alarab, Egypt), Alzental^®^ tablets (EIPICO, 10th of Ramadan City, Egypt) and Curafluke^®^ oral suspension (Univet Ltd., Tullyvin, Ireland) were labeled to contain 500 mg SEC, 10 mg OME, 200 mg ALB, and 50 mg/mL FEN, respectively.

The human plasma was kindly provided by Zagazig University Hospital and was tested to be drug and disease free. Plasma was kept frozen before use, and was then stored either at −4 °C between uses, or at −20 °C for freeze–thaw cycle stability studies.

### 3.3. Procedures

#### 3.3.1. Preparation of Standard Calibration Curves

Aliquot volume from each standard stock solutions was used in 10 mL volumetric flasks to prepare a working standard solution of SEC, OME, ALB, and FEN to acquire a series of concentrations for all drugs (10, 12.50, 25, 50, and 100 µg/mL). A 10 μL of each solution mixture was submitted to HPLC separation and the DAD detector wavelength was set to collect the data at 300 nm. A graph was plotted as concentration of drugs against response (peak area). Three quality control QC samples were prepared for the purpose of method validation at three different concentration (25, 50, and 100 µg/mL) as low (LQC), medium (MQC), and high (HQC) levels, respectively

#### 3.3.2. Pharmaceutical Dosages Procedure

Other than Curafluke^®^oral suspension, 5 tablets of Secnidazole^®^, Omez^®^ and Alzental^®^ tablets were weighed and powdered. An accurately volume or amounts equivalent to 20 mg of each drug were dissolved in the mobile phase, filtered into 100 mL measuring flasks, and completed to volume with the mobile phase. The procedure was then completed as mentioned above, under the general procedure 2.3.1, applying standard addition techniques.

#### 3.3.3. Human Plasma Samples Procedure

Calibration curve and validation QC samples at concentrations of 1.00, 2.50, 5.00, 10.00, and 15.00 µg/mL in plasma were prepared. Into a 10 mL centrifuge tubes, 200 µL of human plasma samples and different drug mixture volumes ranging from 100 up to 200 µL were added followed by 1 min of sample vortex. Methanol was selected as organic solvent to precipitate the human plasma for a total volume 2 Ml. After vortexing for 1 min, the samples were centrifuged at 5000 rpm for 15 min. 10 µL of the supernatant from each sample was filtered through 0.45 µm polytetrafluoroethylene PTFE syringe filters then subjected to HPLC sample analysis.

## 4. Conclusions

The developed HPLC method represents a rapid and simultaneous evaluation of SEC, OME, ALB, and FEN within 5 min. The outcomes achieved reveal that the suggested method is speedy, accurate, selective, robust, and reproducible. Linearity was achieved over a concentration range of 10 to 100 μg/mL for all drugs. The method was effectively useful for the evaluation of advertised formulations Secnidazole^®^, Omez^®^, Alzental^®^ tablets and Curafluke^®^ oral suspension in reverence of quality control, where low-cost and fast analysis are critical. This rational method can be also suitable and valuable for the clinical valuation of SEC, OME, ALB, and FEN in human plasma samples, according to FDA guidelines in respect of pharmacokinetic and bioequivalence studies that would be worthwhile in therapeutic drug monitoring.

## Figures and Tables

**Table 1:** Chromatographic Conditions for the proposed method.

Parameters	Conditions
**Column**	Thermo Scientific^®^BDS Hypersil C_8_ (5 µm, 250 × 4.6 mm)
**Mobile Phase**	Isocratic binary mobile phase of MeOH: 0.025 M KH_2_PO_4_ adjusted to pH 3.20 using ortho- phosphoric acid (70:30, *v/v*), filtered and degassed using 0.45 µm membrane filter
**UV Detection, nm**	300
**Flow Rate, mL/min**	1.20
**Injected Volume, µL**	10.00
**Pressure, psig**	2980
**Temperature**	Ambient

**Table 2:** System suitability parameters for SEC, OME, ALB, and FEN, in both pure and plasma samples.

Parameters	Pure Sample				Plasma Sample				Reference Values [31]
	SEC	OME	ALB	FEN	SEC	OME	ALB	FEN	
**Retention Time, tr**	2.63	3.18	4.03	4.56	2.61	3.14	3.93	4.41	
**Capacity Factor, k’**	1.02	1.45	2.10	2.51	1.01	1.41	2.02	2.39	Accepted k’ value (1–10)
**Peak Asymmetry (Tailing factor, T)**	1.61	1.20	1.05	1.05	0.80	0.76	0.79	0.76	Accepted T value ≤ 2
**TheoreticalPlates, N**	7388	7470	8345	8199	7702	7855	9148	9004	Accepted N value> 2000
**Resolution, Rs**	-----	4.10	5.26	2.79	2.12	4.05	5.16	2.74	Accepted value > 2
**Selectivity (Separation factor, α)**	-----	1.42	1.45	1.19	1.27	1.40	1.43	1.18	

**Table 3:** Analytical merits for determination of SEC, OME, ALB, and FEN in pure samples using the proposed method.

	SEC				OME				ALB				FEN			
	Conc. Taken (µg/mL)	Conc. Found (µg/mL)	Recovery	% Accuracy	Conc. Taken (µg/mL)	Conc. Found (µg/mL)	Recovery	% Accuracy	Conc. Taken (µg/mL)	Conc. Found (µg/mL)	Recovery	% Accuracy	Conc. Taken (µg/mL)	Conc. Found (µg/mL)	Recovery	% Accuracy
	100	100.02	100.02	0.02	100	100.003	100.003	0.003	100	99.86	99.86	−0.13	100	99.87	99.87	−0.12
	50	50.14	100.28	0.28	50	50.09	100.18	0.18	50	50.28	100.57	0.57	50	50.28	100.5	0.56
	25	24.56	98.26	−1.73	25	24.72	98.89	−1.101	25	25.08	100.32	0.32	25	25.05	100.21	0.21
	12.50	12.74	101.95	1.95	12.50	12.62	100.99	0.99	12.50	12.25	98.02	−1.97	12.50	12.26	98.12	−1.87
	10	10.18	101.85	1.85	10	10.05	100.58	0.58	10	10.01	100.13	0.13	10	10.02	100.27	0.27
**Mean**			100.47	0.47			100.13	0.13			99.78	−0.21			99.81	−0.18
**SD**			1.52				0.79				1.01				0.97	
**CV (%)**			1.51				0.78				1.02				0.98	
**SE**			0.67				0.35				0.45				0.43	
**Variance**			2.30				0.62				1.03				0.94	
**Slope**			9.21				17.85				16.47				18.90	
**LOD (µg/mL)**			0.41				0.13				0.18				0.15	
**LOQ (µg/mL)**			1.37				0.44				0.61				0.51	

**Table 4:** Application of standard addition technique for the determination of Secnidazole^®^, Omez^®^, Alzental^®^, and Curafluke^®^ dosage forms using the proposed method.

	SEC (Secnidazole^®^)					OME (Omez^®^)					ALB (Alzental^®^)					FEN (Curafluke^®^)				
	Added Pure Drug (µg/mL)	Taken Tablet (µg/mL)	Conc. Found (µg/mL)	Recovery	% Accuracy	Added Pure Drug (µg/mL)	Taken Tablet (µg/mL)	Conc. Found (µg/mL)	Recovery	% Accuracy	Added Pure Drug (µg/mL)	Taken Tablet (µg/mL)	Conc. Found (µg/mL)	Recovery	% Accuracy	Added Pure Drug (µg/mL)	Taken Tablet (µg/mL)	Conc. Found (µg/mL)	Recovery	% Accuracy
	10	90	101.99	101.99	1.99	10	90	101.93	101.93	1.93	10	90	100.06	100.06	0.06	10	90	101.99	101.99	1.99
	10	40	50.89	101.78	1.78	10	40	49.83	99.67	−0.32	10	40	49.36	98.72	−1.27	10	40	50.19	100.39	0.39
	10	15	25.02	100.10	0.10	10	15	25.41	101.65	1.65	10	15	24.60	98.43	−1.56	10	15	25.32	101.31	1.31
	10	2.50	12.69	101.59	1.59	10	2.50	12.73	101.89	1.89	10	2.50	12.73	101.90	1.90	10	2.50	12.63	101.04	1.04
	10	0.00	9.81	98.13	−1.86	10	0.00	10.08	100.86	0.86	10	0.00	10.18	101.89	1.89	10	0.00	9.98	99.85	−0.14
**Mean**				100.72	0.72				101.20	1.20				100.20	0.20				100.92	0.92
**SD**				1.62					0.95					1.67					0.83	
**CV (%)**				1.61					0.94					1.66					0.82	
**SE**				0.72					0.42					0.74					0.37	
**Variance**				2.65					0.91					2.77					0.68	

**Table 5:** Intra- and inter-day precision results of SEC, OME, ALB, and FEN in pure samples using the proposed method.

	Drugs	Concentrations (µg/mL)	Found Concentration (µg/mL)	Mean Recovery ± SD	CV(%)	% Accuracy
**Intra-day runs** **(n = 3)**	**SEC**	100	99.99	99.99 ± 0.04	0.04	0.01
		50	50.43	100.88 ± 0.02	0.04	0.88
		25	25.03	100.14 ± 0.49	1.96	0.15
	**OME**	100	100.05	100.05 ± 0.07	0.06	0.05
		50	50.27	100.56 ± 0.16	0.33	0.56
		25	24.94	99.79 ± 0.22	0.9	−0.20
	**ALB**	100	100.05	100.05 ± 0.19	0.18	0.05
		50	50.52	101.06 ± 0.21	0.42	1.06
		25	25.4	101.61 ± 0.33	1.3	1.61
	**FEN**	100	100.12	100.12 ± 0.23	0.22	0.12
		50	50.54	101.08 ± 0.22	0.45	1.08
		25	25.33	101.34 ± 0.29	1.14	1.34
**Inter-day runs** **(n = 3)**	**SEC**	100	101.63	101.63 ± 1.40	1.37	1.63
		50	50.96	101.94 ± 0.47	0.93	1.95
		25	24.75	99.03 ± 0.68	2.75	−0.96
	**OME**	100	101.39	101.39 ± 1.21	1.19	1.39
		50	50.96	101.45 ± 0.55	1.08	1.45
		25	24.63	98.56 ± 0.08	0.31	−1.44
	**ALB**	100	101.4	101.40 ± 1.33	1.31	1.41
		50	50.73	101.48 ± 0.39	0.78	1.48
		25	25.02	100.12 ± 0.11	0.44	0.12
	**FEN**	100	101.28	101.28 ± 1.22	1.2	1.28
		50	50.96	101.93 ± 0.59	1.16	1.93
		25	24.99	99.98 ± 0.06	0.24	−0.01

**Table 6:** Results of the robustness for the determination of SEC, OME, ALB, and FEN (50 µg/mL) using the proposed method.

Parameter	SEC			OME			ALB			FEN		
	Mean Recovery ± SD	CV (%)	% Accuracy	Mean Recovery ± SD	CV (%)	% Accuracy	Mean Recovery ± SD	CV (%)	% Accuracy	Mean Recovery ± SD	CV (%)	% Accuracy
**Flow rate 0.95 mL (−0.05)**	101.70 ± 3.03	9.19	1.70	101.60 ± 3.42	11.73	1.60	101.00 ± 3.29	10.88	1.01	101.30 ± 3.67	14.15	1.30
**Flow rate 1.05 mL (+0.05)**	99.53 ± 2.68	7.23	−0.47	99.59 ± 1.41	2.06	−0.41	99.06 ± 1.49	2.22	−0.94	99.46 ± 0.95	0.96	−0.54
**MeOH: Buffer 69.50:30.50 **	100.60 ± 1.52	2.33	0.60	100.57 ± 1.27	1.26	0.57	99.98 ± 1.27	1.63	−0.02	99.84 ± 1.01	1.00	−0.16
**MeOH: Buffer 70.50: 29.50**	100.80 ± 1.65	2.63	0.80	100.61 ± 1.35	1.84	0.61	100.00 ± 1.35	1.83	0.00	99.92 ± 1.11	1.22	−0.08
**Buffer pH 3.15 (−0.05)**	100.70 ± 1.53	2.38	0.30	100.59 ± 1.31	1.73	0.59	100.00 ± 1.31	1.73	0.01	99.90 ± 1.08	1.16	−0.10
**Buffer pH 3.25 (+0.05)**	100.70 ± 1.55	2.44	0.29	100.09 ± 0.79	0.62	0.09	100.10 ± 1.39	1.94	0.10	99.95 ± 1.14	1.29	−0.05

**Table 7:** Statistical analysis of results obtained by the proposed method applied on Secnidazole^®^, Omez^®^, Alzental^®^, and Curafluke^®^ dosage forms compared with reference methods.

	SEC (Secnidazole^®^)		OME (Omez^®^)		ALB (Alzental^®^)		FEN (Curafluke^®^)	
	Proposed Method	Reference Method [10]	Proposed Method	Reference Method [11]	Proposed Method	Reference Method [13]	Proposed Method	Reference Method [13]
**N**	5	6	5	5	5	6	5	6
**Mean Recovery**	100.72	101.00	101.20	100.70	100.20	99.47	100.92	100.20
**SE**	0.72	0.34	0.42	0.38	0.74	0.41	0.37	0.27
**Variance**	2.65	0.71	0.91	0.72	2.77	0.99	0.68	0.43
**Student-t**	0.34 (1.83) ^a^		0.85 (1.86) ^a^		0.90 (1.83) ^a^		1.52 (1.83) ^a^	
**F-test**	3.72 (5.19) ^b^		1.27 (6.39) ^b^		2.78 (5.19) ^b^		1.58 (5.19) ^b^	

^a^ and ^b^ are the Theoretical Student *t*-values and F-ratios at *p* = 0.05.

**Table 8:** Result of analysis of proposed method in human plasma.

	SEC				OME				ALB				FEN			
	Conc. Taken (µg/mL)	Conc. Found (µg/mL)	Recovery	% Accuracy	Conc. Taken (µg/mL)	Conc. Found (µg/mL)	Recovery	% Accuracy	Conc. Taken (µg/mL)	Conc. Found (µg/mL)	Recovery	% Accuracy	Conc. Taken (µg/mL)	Conc. Found (µg/mL)	Recovery	% Accuracy
	15.00	14.60	97.39	−2.60	15.00	14.39	95.95	−4.04	15.00	14.01	93.44	−6.55	15.00	13.22	88.17	−11.82
	10.00	9.46	94.68	−5.31	10.00	9.58	95.88	−4.11	10.00	9.25	92.59	−7.40	10.00	8.70	87.09	−12.90
	5.00	4.94	98.93	−1.06	5.00	4.88	97.67	−2.32	5.00	4.77	95.45	−4.54	5.00	4.47	89.40	−10.59
	2.50	2.37	95.03	−4.96	2.50	2.40	96.28	−3.71	2.50	2.39	95.78	−4.21	2.50	2.16	86.63	−13.36
	1.00	0.95	95.745	−4.25	1.00	0.97	97.07	−2.92	1.00	0.93	93.51	−6.48	1.00	0.87	87.55	−12.44
**Mean**			96.35	−3.64			96.57	−3.42			94.15	−5.84			87.77	−12.22
**SD**			1.77				0.77				1.38				1.07	
**CV (%)**			1.84				0.80				1.47				1.22	
**SE**			0.79				0.34				0.62				0.47	
**Variance**			3.16				0.59				1.92				1.15	

**Table 9:** Stability results of SEC, OME, ALB, and FEN in plasma samples using the proposed method.

	Drugs	Concentrations (µg/mL)	Found Concentration (µg/mL)	Mean Recovery ± SD	CV (%)	% Accuracy
**After 24 h At Ambient Temperature** **(N Plasma = 3)**	**SEC**	15	14.72	98.18 ± 0.68	4.66	−1.80
		5	4.97	99.52 ± 0.03	0.58	−0.47
		1	0.99	99.88 ± 0.03	3.55	−0.11
	**OME**	15	14.4	96.01 ± 0.06	0.45	−3.97
		5	4.84	97.01 ± 0.02	0.6	−2.98
		1	0.96	96.74 ± 0.01	0.29	−3.24
	**ALB**	15	14.01	93.38 ± 0.04	0.33	−6.60
		5	4.73	94.73 ± 0.03	0.73	−5.26
		1	0.93	93.87 ± 0.01	0.33	−6.12
	**FEN**	15	13.25	88.37 ± 0.17	1.3	−11.62
		5	4.45	89.21 ± 0.01	0.26	−10.77
		1	0.87	87.24 ± 0.01	0.01	−12.74
**3 Freeze–Thaw Cycles At −20 °C (N Plasma = 3)**	**SEC**	15	14.84	98.97 ± 1.80	12.17	−1.02
		5	5.06	101.29 ± 0.16	3.28	1.3
		1	1.03	103.72 ± 0.09	8.64	3.72
	**OME**	15	14.35	95.71 ± 0.59	4.1	−4.28
		5	4.83	96.68 ± 0.05	1.03	−3.31
		1	0.98	98.39 ± 0.02	2.75	−1.59
	**ALB**	15	14.02	93.44 ± 0.09	0.64	−6.54
		5	4.77	95.45 ± 0.05	1.15	−4.53
		1	0.95	95.67 ± 0.03	3.17	−4.32
	**FEN**	15	13.26	88.44 ± 0.26	1.96	−11.54
		5	4.47	89.61 ± 0.03	0.68	−10.37
		1	0.89	89.70 ± 0.04	4.52	−10.28

## Sample Availability

Samples of the compounds are not available from the authors.
